# Unlocking Sustainable‐by‐Design Li‐Metal Batteries by Recycled PVB in Blend Polymer Electrolytes

**DOI:** 10.1002/cssc.202501288

**Published:** 2025-08-18

**Authors:** Asia Patriarchi, Hamideh Darjazi, Alessandro Piovano, Leonardo Balducci, Nicolò Arcieri, Miguel Ángel Muñoz‐Márquez, Francesco Nobili, Claudio Gerbaldi

**Affiliations:** ^1^ School of Science and Technology Chemistry division University of Camerino Via Madonna delle Carceri‐ChIP 62032 Camerino (MC) Italy; ^2^ Department of Applied Science and Technology Politecnico di Torino Corso Duca degli Abruzzi, 24 10129 Torino Italy; ^3^ National Reference Center for Electrochemical Energy Storage (GISEL)‐INSTM Via G. Giusti 9 50121 Firenze Italy

**Keywords:** lithium batteries, poly(ethylene oxide), poly(vinyl butyral), polymer electrolyte, recycling, solid‐state batteries, solvent‐free

## Abstract

In today's sustainability‐driven society, the circular economy has emerged as a guiding principle for responsible resource use, aiming to transform production by prioritizing reuse, repair, and recycling, thus extending product lifecycles and reducing waste. Herein, the impact of the circular economy on upcycling, focusing on poly(vinyl butyral) (PVB), widely used as an interlayer in laminated glass, recovered from automotive waste via a patented mechanochemical process, is investigated. For the first time, this process is applied in an innovative closed‐loop design for solid polymer electrolytes (SPEs) in Li–metal batteries (LMBs). A green, solvent‐free method produces polymer blends of PVB, ensuring outstanding mechanical properties, and poly(ethylene oxide) (PEO) as the ion‐conductive matrix. PVB effectively reduces PEO crystallinity, improving ionic conductivity, mechanical strength, and oxidative stability. The resulting SPEs exhibit stable lithium stripping/plating and reduced interfacial resistance in symmetric cells, confirming excellent lithium compatibility. Lab‐scale lithium metal polymer cells with a high‐loading LiFePO_4_‐based catholyte (13 mg cm^−^^2^) achieve near‐theoretical capacities at low C‐rates (154.4 mAh g^−1^ at C/5) and excellent rate performance at 65 °C. By promoting a holistic approach to sustainable resource use, PVB contributes to the development of high‐performance, environmentally sustainable polymer electrolytes for next‐generation LMBs.

## Graphical abstract

Recovered poly(vinyl butyral) (PVB) from automotive glass is upcycled via a patented mechanochemical process into solid polymer electrolytes for Li‐metal batteries. A green, solvent‐free method yields PVB–poly(ethylene oxide) blends with enhanced conductivity, stability, and lithium compatibility, enabling high‐capacity, high‐rate performance and promoting circular economy principles in sustainable, next‐generation energy storage.

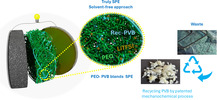

## 1 Introduction

Rapid population growth, industrialization, and urbanization have led to critical levels of waste generation, threatening ecosystems and human health. Effective waste management has become a global priority, especially as municipal solid waste is projected to rise from 2.01 to 3.40 billion tons by 2050. In low‐income regions, up to 90% of waste is still burned or dumped, worsening environmental and health risks. To address this, the circular economy model, being focused on conserving resources and reusing materials in a closed‐loop approach, is gaining momentum.^[^ ^1^ ^]^ Yet, limited recycling infrastructure hampers full implementation. Converting waste into high‐value products presents a promising, though still challenging, solution.^[^ ^2^ ^]^ Meanwhile, growing energy demand and fossil fuel depletion are driving the shift to cleaner technologies. Electric vehicles (EVs), central to this transition, depend heavily on the safety, reliability, and performance of their electrochemical energy storage (viz., battery) systems.^[^ ^3^, ^4^, ^5^, ^6^ ^]^

Among various energy storage technologies, lithium‐ion batteries (LIBs) have emerged as a leading solution, offering high energy density, long cycle life, and efficient storage capabilities.^[^ ^7^, ^8^ ^]^ However, from the perspectives of safety and sustainability (e.g., flammability, leakage risks, and thermal instability), as well as practical recycling, conventional LIBs with liquid electrolytes exhibit significant limitations, fostering the search for greener processes and innovative materials.^[^ ^9^, ^10^, ^11^ ^]^ In this context, solid polymer electrolytes (SPEs) have emerged as a safer alternative, assuring enhanced mechanical support to the battery and improved energy density. Furthermore, SPEs provide better thermal stability and feature an eco‐friendly composition, by eliminating the need for toxic and flammable organic solvents, while also holding the promise of easier recycling at the end‐of‐life.^[^ ^12^, ^13^, ^14^ ^]^

Among all the polymer matrices that have been evaluated as potential candidates for SPEs, which include poly(vinylidene fluoride) (PVDF),^[^ ^15^, ^16^ ^]^ poly(ethylene glycol) (PEG),^[^ ^17^ ^]^ poly(vinyl alcohol) (PVA),^[^ ^18^ ^]^ and poly(methyl methacrylate) (PMMA),^[^ ^19^ ^]^ poly(ethylene oxide) (PEO) is particularly attractive due to its excellent processability (also in dry processing, e.g., extrusion), elastic behavior, and strong interfacial compatibility with lithium metal anodes. The ionic conduction mechanism in PEO relies on the mobility of its polymer chains, where a lower degree of crystallinity facilitates faster Li^+^ transport by enhancing chain segment motion.^[^ ^20^, ^21^, ^22^ ^]^ Nevertheless, increasing the flexibility of the polymer matrix typically results in a reduction of mechanical strength. Indeed, like most SPE systems, PEO‐based SPEs face an inherent trade‐off between achieving high ionic conductivity and maintaining adequate mechanical integrity, making it challenging to optimize both characteristics simultaneously.^[^ ^23^, ^24^, ^25^, ^26^, ^27^ ^]^ Temperature also plays a crucial role in influencing polymer behavior.^[^ ^28^ ^]^ Elevated temperatures promote greater segmental motion and faster ion transport, but they simultaneously weaken the mechanical framework, sometimes softening the PEO matrix to a near‐molten state that increases the risk of internal short circuits. The longstanding challenge of balancing conductivity and mechanical strength remains a major barrier to the broader application of SPEs, particularly at low (ambient and below) temperature conditions.

In this regard, blending with polymers with strong mechanical properties without negatively affecting ionic mobility is driving the practical development of high‐performing SPE in advanced solid‐state batteries. Poly(vinyl butyral) (PVB), a versatile polymer traditionally used as an interlayer in laminated safety glass such as in car windshields and building windows, is a promising candidate. PVB offers excellent mechanical support, hardness, and superior film‐forming ability.^[^ ^29^, ^30^ ^]^ However, due to the deterioration of the PVB optical properties upon glass reprocessing (thus not meeting anymore the glass industry's quality standards, PVB is often discarded during glass recycling processes, creating an underutilized waste stream.^[^ ^31^, ^32^, ^33^ ^]^ Recovering and repurposing PVB from laminated glass waste into the electrochemical field aligns well with the principles of a circular economy promoting sustainability.^[^ ^34^, ^35^ ^]^ The European “SUNRISE” project directly addresses this need by focusing: 1) on the selective recovery of the PVB fraction that can be recycled for its initial use, and 2) the repurposing/upcycling of its nonrecyclable fraction into high‐value applications, such as in energy storage devices.

Few reports are available in the literature on the use of PVB in LIBs, including it as a component in separators,^[^ ^36^ ^]^ binders,^[^ ^37^ ^]^ and gel polymer electrolytes.^[^ ^38^ ^]^ However, to best of our knowledge, its use in combination with PEO as a true SPE has not been explored yet. Even more remarkably, the incorporation of the nonrecyclable fraction of PVB (Rec PVB) into PEO‐based SPE could address both performance and sustainability challenges, allowing the design of a composite polymer electrolyte with enhanced mechanical strength, thermal stability, and electrochemical performance, while simultaneously promoting the use of recycled materials to reduce environmental impact, well in agreement with the circular economy model previously discussed.

The physical and electrochemical properties of novel PEO‐based SPEs are investigated by incorporating varying concentrations of commercial PVB (3, 5, and 10%) and different molar ratios of EO to Li, specifically 21:1 and 15:1. After evaluating these formulations, the most promising composition is identified, and commercial PVB is subsequently replaced with Rec PVB sourced from automotive glass waste via a patented process.^[^ ^39^ ^]^ The resulting SPE is then thoroughly characterized to assess its overall performance. In addition to focusing on sustainability through the use of recycled materials and enhancing battery performance, particular emphasis is placed on the preparation method (solvent‐free) and the incorporation of a high‐loading cathode, both of which contribute to improving the practical applicability and scalability of the developed SPE for lithium‐metal batteries.

## 2 Results and Discussion

### 2.1 Structure Design and Characterization of PEO/PVB SPEs

**Figure 1:**
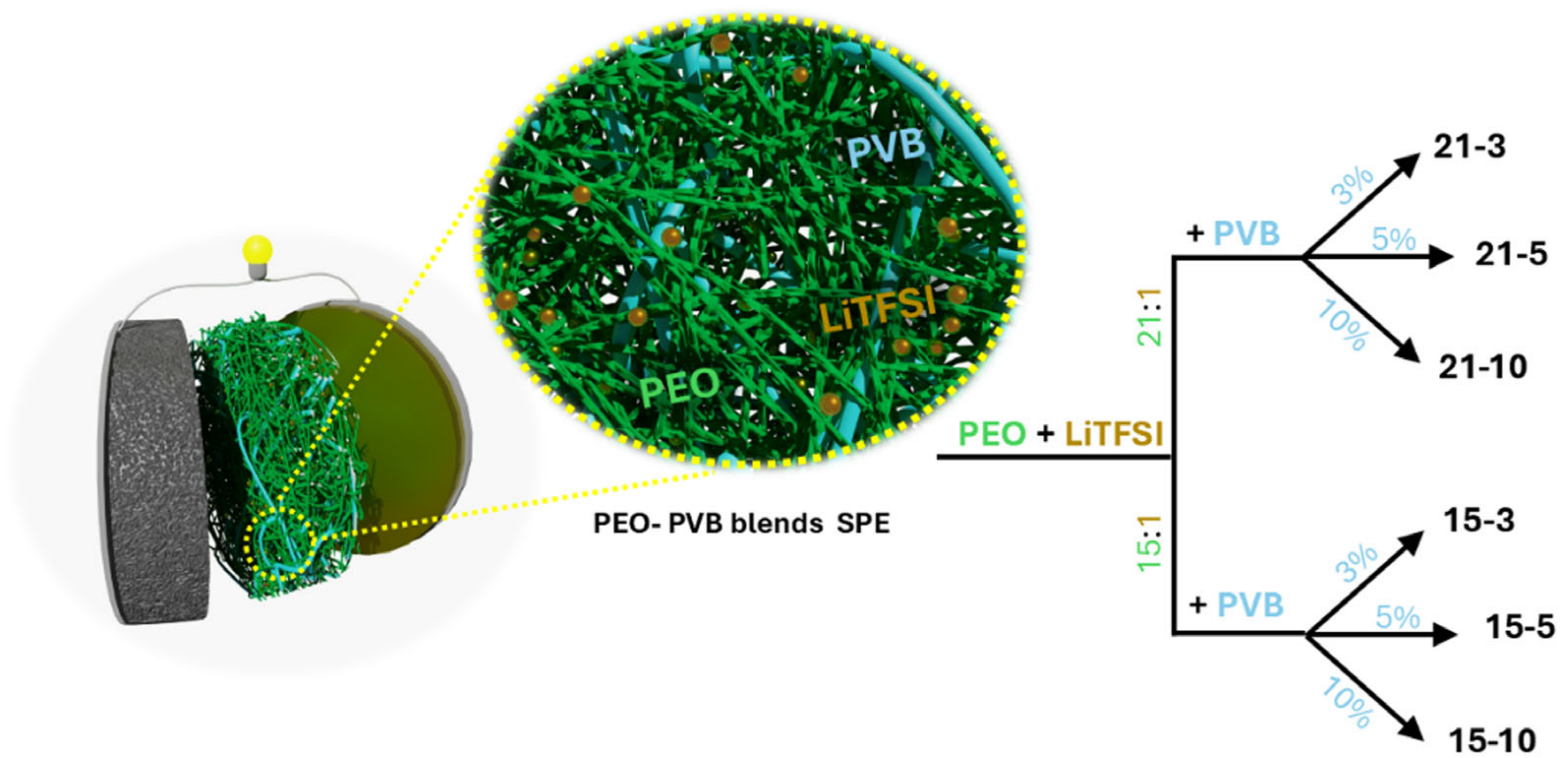
Schematic representation of the synthesized electrolyte with different EO/Li ratio and PVB content.

Truly SPEs were prepared using a solvent‐free method, with a focus on potential scalability, to optimize the formulation in view of using of Rec PVB. These electrolytes were prepared following the protocol described in the Experimental Section, and they are hereafter designed depending on their specific composition, EO/LiTFSI molar ratio, and varying commercial PVB content—namely, 21‐3, 21‐5, 21‐10 (Series 21), 15‐3, 15‐5, and 15‐10 (Series 15), as illustrated in **Figure** **1**.

**Figure 2:**
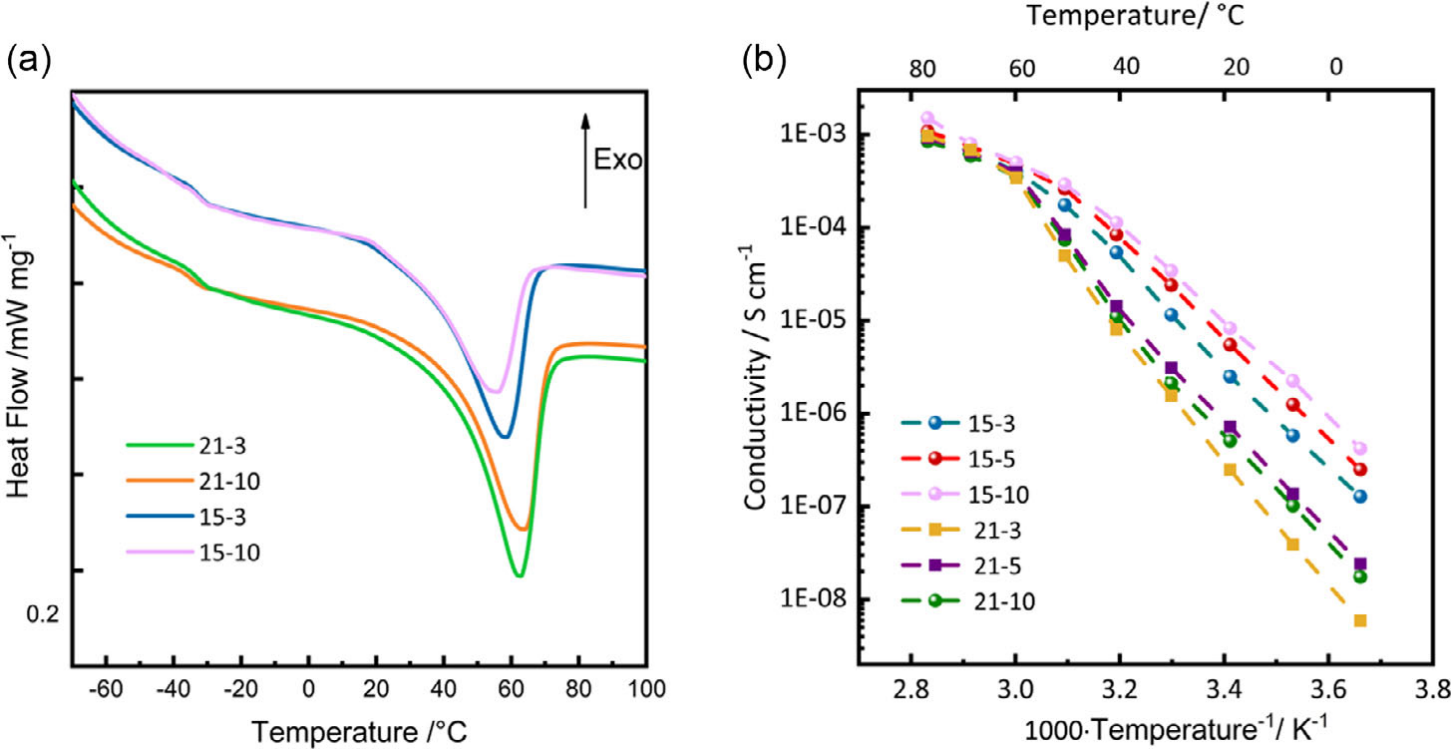
a) DSC thermograms and b) Arrhenius plots of ionic conductivity versus inverse temperature determined by EIS of the different SPEs under study.

Differential scanning calorimetry (DSC) was conducted to evaluate the thermal behavior of the SPEs (**Figure** **2**a). Whereas the T_g_ is almost the same for all the samples (at about −32 °C), the melting point (T_m_) is visibly influenced by the EO:Li ratio, with the membranes of the Series 21 melting at ≈62 °C and those of the Series 15 melting at ≈57 °C. As a matter of fact, the salt displays a plasticizing effect: the higher is the salt loading, the lower is T_m_ with respect to pure PEO. Besides, the different DSC profiles give an information about the crystallinity degree of the material, derived from the heat exchanged per gram of sample. This is an important parameter to be evaluated in SPEs since it is widely reported in the literature that alkali metal ions migrate much faster in amorphous PEO regions with respect to crystalline ones, which results in enhanced ionic conductivity.^[^ ^40^ ^]^ In this respect, from the analysis of the data in Figure 2a, it is possible to conclude that increasing the proportion of PVB from 3 to 10% leads to a progressive decrease in the crystallinity degree (from 30% to 23% for the Series 21, and from 25% to 17% for the Series 15), indicating enhanced disruption of the polymer ordered regions.

Ionic conductivity was also measured for all samples across a temperature range of 0 to 80 °C to assess the influence of PVB incorporation and lithium salt concentration on the transport properties of the SPE (Figure 2b). It is worth noting that the incorporation of PVB significantly influences the ionic conductivity, resulting in a better segmental mobility of the polymer chains and facilitated ion transport compared to the pristine electrolyte (without PVB)^[^ ^41^ ^]^ As shown in Figure 2b, all SPEs exhibit a distinct change in slope around 60 °C, corresponding to the melting of their crystalline regions—most notably in the Series 21 (21‐3, 21‐5, and 21‐10).^[^ ^42^, ^43^ ^]^ In contrast, Series 15 (15‐3, 15‐5, and 15‐10) display a less abrupt slope change, with a more gradual curve. These observations suggest a decrease in the crystalline domains as the EO:Li ratio changes from 21:1 to 15:1. In particular, higher concentration of LiTFSI has been reported to promote the formation of more amorphous domain with enhanced chain mobility, causing a decreasing of matrix stiffness.^[^ ^44^ ^]^ This is further supported by the Arrhenius plot, where the Series 15 exhibit slightly higher ionic conductivity. In particular, the 15‐10 SPE shows remarkable performance, with conductivities up to 7.9 × 10^−^^4^ and 3.4 × 10^−5^ S cm^−1^ at 70 and 30 °C, respectively. This enhancement is likely due to facilitated Li^+^ ion mobility by coupling/decoupling and lithium‐ion hopping mechanism, in which Li^+^ ions are reversibly bonded to the oxygen atoms in the polymer matrix.^[^ ^45^ ^]^ Additionally, improved conductivity can be attributed to the enhanced ability of the15‐10 SPE to dissolve lithium salts without forming stable complexes that would otherwise hinder polymer chain mobility.

**Figure 3:**
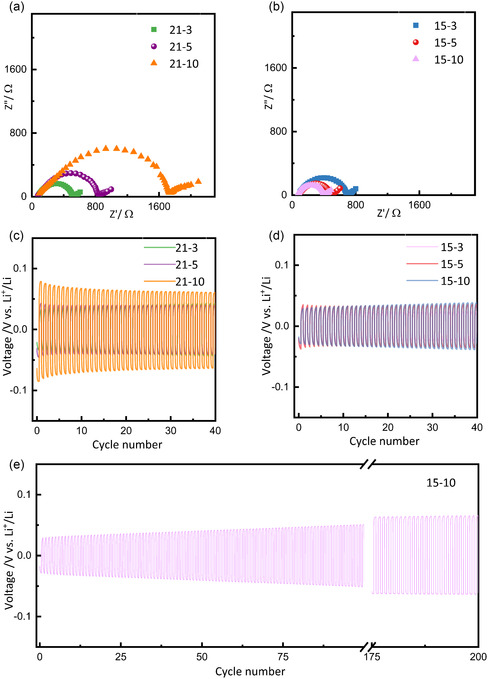
Nyquist plots and lithium stripping/plating tests recorded at 65 °C in Li||SPE||Li symmetric cells for a,c) 21‐3, 21‐5, and 21‐10 SPEs and b,d) 15‐3, 15‐5, and 15‐10 SPEs. e) Long‐term stripping/plating of the 15‐10 SPE.

The electrolytes were evaluated in a Li||SPE||Li symmetric cell configuration at 65 °C to assess their electrochemical performance. Electrochemical impedance spectroscopy (EIS) analysis reveals two distinct trends depending on the polymer‐to‐salt ratio. For the Series 21 membranes (**Figure** **3**a), increasing the PVB concentration from 3 to 10% led to a noticeable rise in overall resistance. This increase can be attributed to the added rigidity introduced by PVB, which likely restricts the mobility of the polymer chains and reduces segmental motion essential for Li^+^ ion conduction.^[^ ^46^, ^47^ ^]^ The hindered segmental dynamics disrupts the lithium hopping mechanism, leading to the formation of a stiffer matrix that hinders efficient ion conduction and ultimately compromises the overall electrochemical performance. Correspondingly, the electrolyte resistance (R_e_) shows a progressive increase, with values of 77, 89, and 91 Ω, for the 21‐3, 21‐5, and 21‐10, respectively.

Conversely, for the Series 15 SPEs (Figure 3b), increasing the PVB concentration has a beneficial effect, significantly reducing the overall resistance. In this case, the higher concentration of LiTFSI introduces a plasticizing effect, associated with a greater number of mobile Li^+^ ions, which enhances ion migration and mobility throughout the electrolyte.^[^ ^4^ ^]^ However, this increase in salt content may simultaneously weaken the structural integrity of the polymer network, making the SPE more prone to deformation and dendrite formation. Notably, the addition of PVB at this ratio plays a crucial mechanical role. Its incorporation strengthens the polymer matrix, improving resistance to mechanical stress and preserving structural stability. This mechanical reinforcement counterbalances the structural softening caused by the plasticizing salt; indeed, nanoindentation experiment revealed a reduced Young's modulus of 10 MPa (Figure S3, Supporting Information), significantly higher than the value of 2.95 MPa reported by Fu et al. for pristine PEO‐LiTFSI membrane^[^ ^48^ ^]^ Overall, the favorable trend of R_e_ values in Figure 3b (decreasing from 93, 90, and 72 Ω for the 15‐3, 15‐5, and 15‐10 membranes, respectively) confirms the optimal balance between enhanced ionic conductivity and mechanical robustness within the Series 15 SPEs.^[^ ^49^, ^50^, ^51^, ^52^ ^]^

The stripping and plating test shows higher overpotentials of 0.032, 0.042, and 0.078 V for 21‐3, 21‐5, and 21‐10 SPEs (see Figure 3c) compared to 0.038 V, 0.035 V, and 0.023 V for 15‐3, 15‐5, and 15‐10 SPEs (see Figure 3d). This trend is consistent with the higher resistance values observed in the EIS analysis for the 21 series. Among all the tested electrolytes, 15‐10 SPE exhibits the lowest overpotential, achieved after a few activation cycles, likely necessary to establish an optimal Li‐electrolyte interface. The formation of a stable interface significantly improves charge transfer kinetics, resulting in lower overpotential and enhanced electrochemical performance. Moreover, 15‐10 SPE (Figure 3e) demonstrates exceptional stability during prolonged cycling in symmetrical Li||SPE||Li cell, with no signs of dendrite formation or cell failure, in contrast to pristine PEO without PVB, as shown in Figure S1a,b, Supporting Information.

As a result, the optimization of the electrolyte formulation, achieved by adjusting the EO to lithium salt molar ratio to 15:1 and incorporating 10 wt% of commercial PVB, led to the development of a high‐performance solid polymer electrolyte (referred to as 15‐10). The addition of PVB partially disrupted the crystalline domains of the PEO matrix, thereby enhancing the proportion of amorphous regions, which are crucial for efficient ion transport. Furthermore, the optimized polymer–salt ratio facilitated better polymer chain segmental mobility and ion dissociation, resulting in lower interfacial resistance and superior electrochemical performance, highlighting the beneficial role of PVB in the electrolyte formulation.

### 2.2 Characterization of PEO/Rec PVB

Given the global push toward sustainable materials and the growing interest in using recycled polymers as substitutes for commercial counterparts, we first prepared a truly SPE by incorporating Rec PVB, sourced from automotive glass waste through a patented mechanochemical process. This recycled polymer was integrated into the formulation as a copolymer, to create an eco‐friendly and cost‐effective alternative. Building on the results obtained with commercial PVB and shown above, the 15‐10 composition was identified as the optimal electrolyte formulation and used as the baseline for further optimization with Rec PVB. The synthetic protocol was then adapted to substitute the commercial PVB with the recycled variant, as illustrated in Figure S2a, Supporting Information, denoted as 15‐Rec10. Prior studies have characterized this Rec PVB using Fourier transform infrared (FT‐IR), thermogravimetric analysis (TGA), and nuclear magnetic resonance (NMR), demonstrating that the mechanochemical recycling process does not alter the polymer molecular weight (≈135 000 g mol^−1^, with a broad distribution). These studies also identified the presence of various plasticizers, all belonging to the diester class, with an estimated polymer‐to‐plasticizer weight ratio of about 1:1.^[^ ^39^ ^]^

The obtained SPE was studied using scanning electron microscopy (SEM) microscopy (to ensure adequate electronic conductivity, a metallization step was performed prior to the analysis). The SEM image in Figure S2b, Supporting Information shows a smooth, defect‐free surface, without visible cracks or significant roughness. Achieving a good surface uniformity is particularly important, as the presence of structural imperfections could negatively impact the electrolyte‐electrode interface, potentially reducing surface contact area and hindering ion diffusion.

The crystalline structure of 15‐Rec10 was examined using X‐ray diffraction (XRD), as illustrated in Figure S2c, Supporting Information. The XRD diffractogram confirms the semicrystalline nature of the prepared electrolyte. Notably, two sharp and intense peaks at 19.2° and 23.4° are clearly visible, which are characteristic of the crystalline domains of PEO.^[^ ^53^ ^]^ However, the addition of significant amount of LiTFSI induces a pronounced plasticizing effect, as evidenced by the broadening of these peaks which arises from the coordination of lithium ions with the oxygen atoms in polyethylene oxide, leading to a reduction in crystallinity compared to previously reported diffractograms of pure PEO.^[^ ^54^, ^55^, ^56^ ^]^ Additionally, the XRD pattern reveals a well‐defined overlap with the characteristic broad peaks of PVB at 19° and 42.5°, which significantly contributes to the overall amorphous nature of the material.^[^ ^57^ ^]^ Also for SPE including Rec PVB, the combination of crystalline and amorphous domains is expected to provide a balance between mechanical stability and ionic mobility in the electrolyte.

The thermal stability of 15‐Rec10 was assessed by TGA, in the 30 to 550 °C range under nitrogen flow (Figure S2d, Supporting Information). The first event, accounting for ≈4% of the total mass, occurs between 70 and 150 °C, and described an initial loss likely due to the evaporation of adsorbed water or moisture at the membrane surface, which is a result of the sample hygroscopic nature. The second weight‐loss event, occurring between 300 and 350 °C, corresponds to the loss of plasticizers from Rec PVB, followed by the onset of polymeric matrix decomposition. During this stage, the PEO chains undergo random scission, leading to the fragmentation of the polymer into smaller molecular products.^[^ ^47^ ^]^ Simultaneously, PVB degrades, with the decomposition of its butenal and butanol groups, followed by the elimination of butyral groups.^[^ ^58^, ^59^ ^]^ The final decomposition stage, observed between 425 and 460 °C, involves the carbonization of both polymers. At this stage, the organic matrix is converted into carbon, resulting in a significant weight loss of ≈80%. Despite these decomposition processes, the electrolyte demonstrates excellent thermal stability, remaining intact up to 300 °C, which underscores its suitability for applications requiring high thermal resistance.

**Figure 4:**
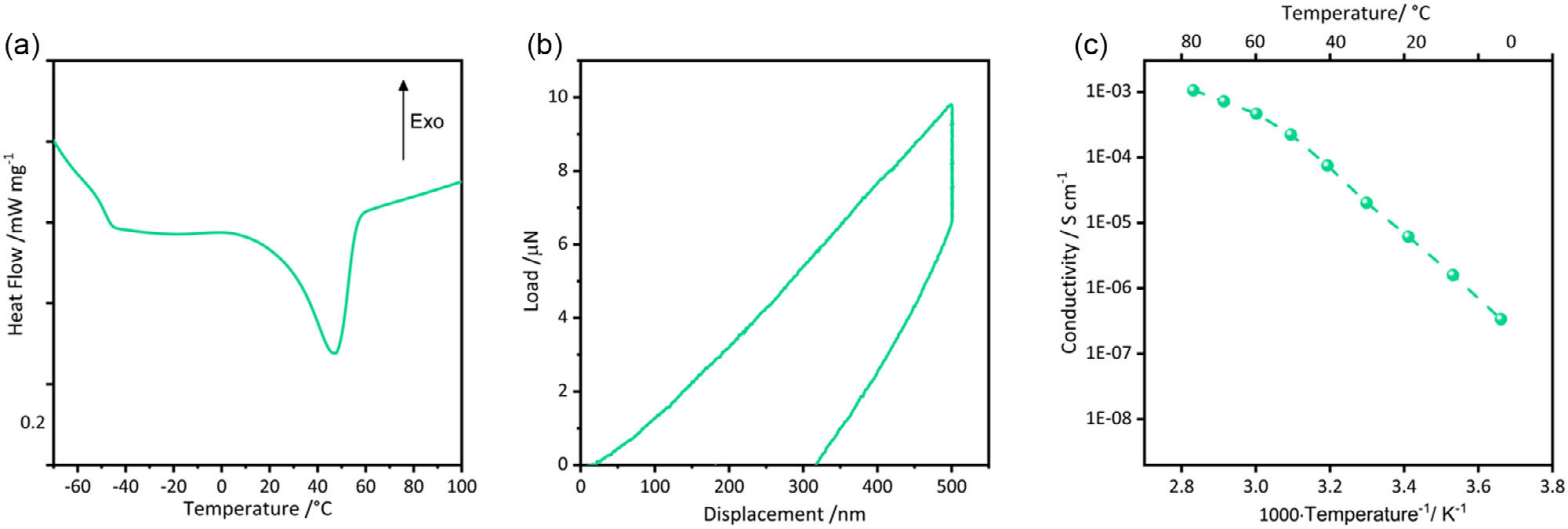
a) DSC thermogram, b) representative nanoindentation curve, and c) Arrhenius plots of ionic conductivity versus inverse temperature determined by EIS of the 15‐Rec10 membrane.

The thermal behavior of the 15‐Rec10 membrane is evaluated by DSC analysis, as shown in **Figure** **4**a, revealing a T_m_ shift to 46 °C and a T_g_ shift to –47 °C. Notably, the very low T_g_, likely due to the presence of residual plasticizers in the Rec PVB, promotes extensive segmental motion of the polymer chains, which compensates for the slightly higher degree of crystallinity (20%).^[^ ^60^ ^]^

To assess the impact of PVB, particularly Rec PVB, on PEO/PVB blend SPEs compared to pristine PEO electrolyte, mechanical strength was evaluated. As expected, considering that Rec PVB was sourced from laminated safety glasses (where its role was, indeed, to withhold the glass fragments upon accidental cracks), 15‐Rec10 SPE displays an even higher reduced Young's modulus upon nanoindentation test (21 MPa) than 15‐10 membrane, enduring up to 10 μN of load at a penetration depth of 0.5 μm (as shown in Figure 4b). This is a promising result in the perspective of exploiting 15‐Rec10 as SPE into a Li‐metal electrochemical cell, where SPEs exert the double role of medium for Li^+^ ions transport and of barrier against Li dendrites growth.

The ionic conductivity of the membrane containing Rec PVB (Figure 4c) is also comparable to that of the membrane with commercial PVB of the same composition (15‐10, Figure 2b). Thus, a simultaneous enhancement of ionic conductivity and mechanical strength in SPEs was achieved by developing an electrolyte based on Rec PVB combined with ion‐conductive PEO.

The Li^+^ transference number (t_Li+_) reflects the proportion of charge carried by lithium ions and plays a crucial role in controlling the deposition morphology of lithium metal. The t_Li+_ of the 15‐10 electrolyte was evaluated at 55 and 65 °C using the Bruce–Vincent–Evans method, as detailed in the Experimental Section. The obtained t_Li+_ values are 0.19 and 0.22, respectively, with a slight increase observed at the higher temperature. This improvement can be attributed to enhanced lithium‐ion solvation, facilitated by the increased flexibility of the polymeric matrix above the melting point. These values align well with those previously reported for similar PEO‐based polymer electrolytes.^[^ ^61^, ^62^, ^63^ ^]^

**Figure 5:**
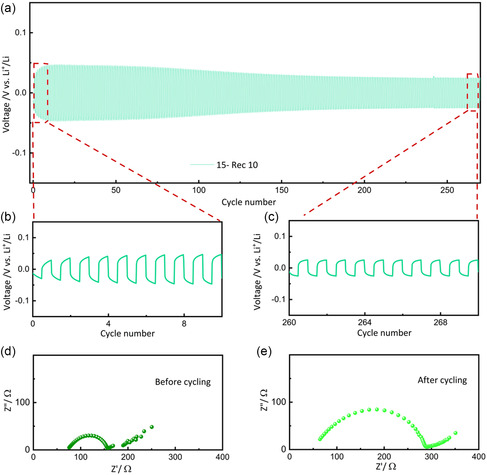
a) Long‐term lithium stripping/plating galvanostatic cycling test performed on a Li||15‐Rec10||Li symmetric cell at 65 °C. Magnified views of the stripping/plating profiles between b) cycles 0–10 and c) cycles 260–270. d) EIS spectra obtained at OCV and e) after 270 cycles.

The electrochemical performance of the electrolyte was also assessed in a Li||15‐Rec10||Li symmetric cell configuration at 65 °C, as shown in **Figure** **5**a. The stripping and plating profiles demonstrate a remarkable stability over more than 250 cycles, with no signs of degradation and/ or dendrite formation. A few activation cycles were necessary to achieve optimal performance, as evidenced in Figure 5b, which provides a detailed view of the first ten stripping and plating cycles. During these initial cycles, the overpotential increased to ≈0.047 V, followed by a gradual decrease. This behavior is likely attributed to the complete thermal activation of the SPE, which may enhance lithium‐ion mobility by reducing the crystallinity of the polymer matrix. While this lower‐quality PVB is unsuitable for use in the glass industry, it could offer significant advantages for electrolyte formulations. The cycling stability of 15‐Rec10 is particularly promising, with an overpotential value of ≈0.025 V after 260 cycles (Figure 5c), which is less than half of the value observed after 200 cycles for the analogous SPE containing commercial PVB (0.06 V), as shown in Figure 3e. In addition, stripping/plating tests were conducted at various current densities ranging from 0.05 to 1 mA cm^−2^ (total capacity: 0.1 mAh cm^−^^2^), displaying stable cycling at all current densities, and, remarkably good rate capability, with rather limited overpotential even at very high (for a truly solid‐state polymer electrolyte) current regime of 1 mA cm^−2^; results are shown in Figure S4, Supporting Information. The Nyquist plots recorded before and after the stripping and plating tests are shown in Figure 5d,e, respectively. Prior to cycling, 15‐Rec 10 demonstrates a low overall resistance of 156 Ω, outperforming its commercial counterpart (405 Ω) and indicating a reduced lithium‐ion interfacial polarization enabled by the incorporation of Rec PVB. Additionally, the R_e_ (65 Ω) is slightly lower than that of commercial 15‐10 (72 Ω), which is in good agreement with earlier findings. After cycling, the R_e_ value further decreases to 53 Ω, indicating improved membrane activation and enhanced lithium‐ion mobility, in line with the stripping and plating results. However, a slight increase in the overall resistance is observed, likely due to the formation of a passivation layer during the initial cycles, which may partially consume the Li salt and contribute to the polarization increase.

### 2.3 Performances of PEO/Rec PVB in Lithium‐Metal Batteries

The electrochemical stability of the developed SPE was initially evaluated to assess their suitability as SPEs in Li‐metal cells. LSV was applied to determine the onset potential of electrolyte degradation at anodic oxidation potential. As shown in Figure S5a, Supporting Information, the 15‐Rec10 sample demonstrates enhanced oxidative stability, sustaining a stable current response up to ≈4.58 V—which is comparable with the pristine electrolyte (Figure S1c, Supporting Information), based on a current threshold of 5 μA cm^−^^2^.

To further define the stability limits, chronoamperometry measurements were conducted, applying a fixed potential for one hour at each step. The 15‐Rec10 sample (Figure S5b, Supporting Information) maintains a rather stable current profile for one hour at 4.5 V, while at 4.6 V, some current spikes are observed after about 30 min, marking the onset of degradation. This is better compared to the pristine electrolyte, where degradation begins at 4.5 V (Figure S1d, Supporting Information), as indicated by a gradual rise in current, with complete breakdown occurring at 4.6 V. It suggests that the incorporation of Rec PVB slightly improves the oxidative stability of the electrolyte, extending its electrochemical stability window (ESW) relative to the pristine formulation.

**Figure 6:**
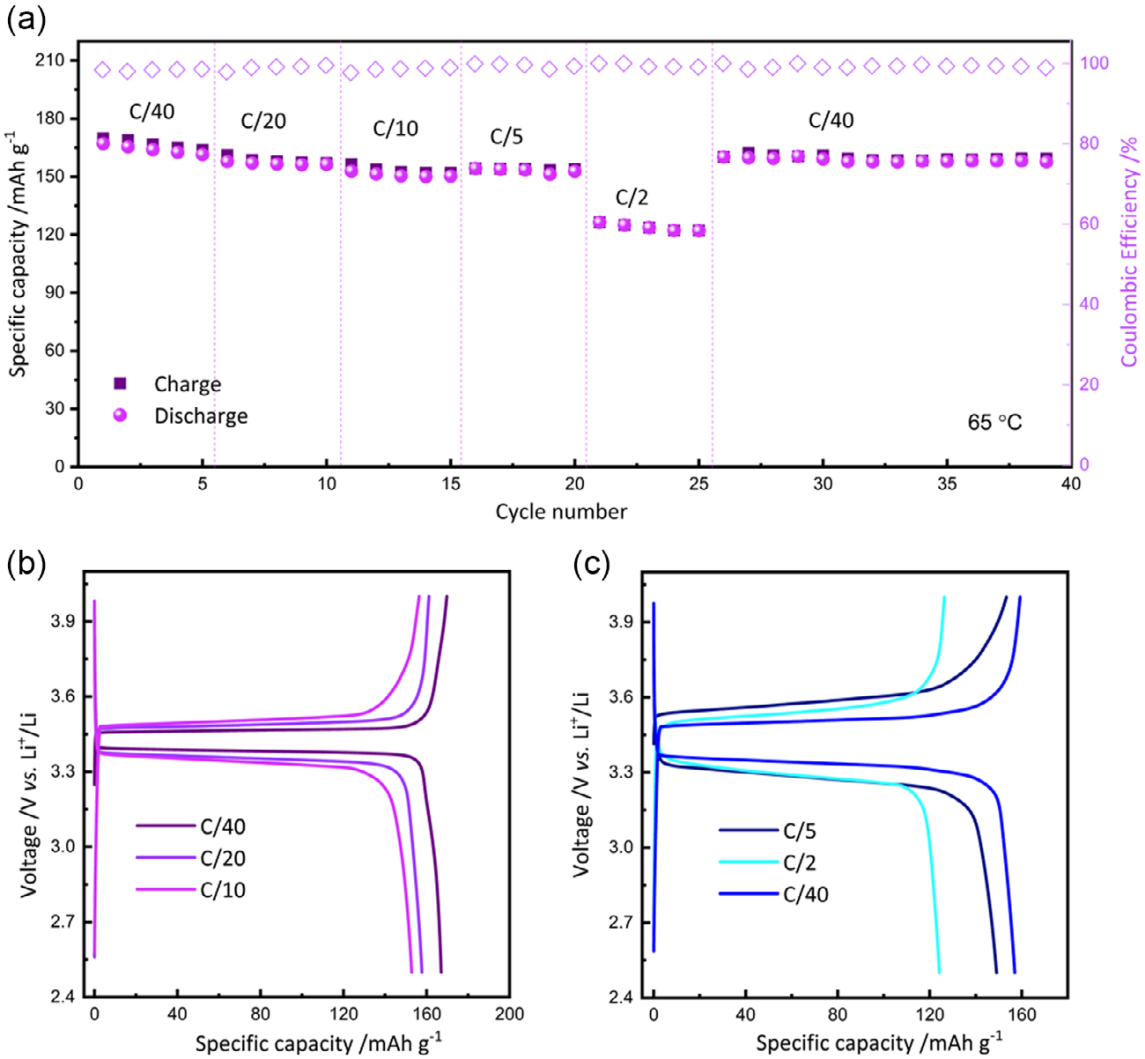
a) Rate capability of the Li||15‐Rec 10||LFP at 65 °C and b,c) the corresponding voltage distribution curves of charge and discharge.

The charge/discharge performance of 15‐Rec10 in truly solid‐state Li‐metal cells was evaluated using an LFP‐based electrode in a high‐loading catholyte configuration (13 mg cm^−^^2^). **Figure** **6**a shows the capacity as a function of current density at C/40, C/20, C/10, C/5, C/2, and back to C/40 within the voltage range of 2.5–4.0 V vs. Li^+^/Li. During the initial cycle at C/40, the cell achieves almost full capacity of 169.8 mAh g^−1^ with a Coulombic efficiency (CE) of 98.4%. Additionally, high capacities of 161.2, 156.4, 154.4, and 126.3 mAh g^−1^ were maintained at C/20, C/10, C/5, and C/2, respectively, with a CE of 99%. These findings are in line with state‐of‐art SPE‐based LFP cells reported in recent literature (as resumed in Table S1),^[^ ^64^, ^65^, ^66^, ^67^, ^68^ ^]^ demonstrating the excellent rate capability of the 15‐Rec10‐based truly solid electrochemical cell, across all tested C‐rates, preserving high specific charge capacity despite increasing current. Furthermore, as shown in Figure 6b,c, the polarization voltage remains minimal across varying current densities, even at higher C‐rates, confirming promising cycling stability. The superior rate performance of the Li||15‐Rec10||LFP cell with a high‐loading cathode can be attributed to the high Li^+^ conductivity of SPE and its robust electrode‐electrolyte compatibility, facilitating uniform Li^+^ deposition and stable solid electrolyte interphase layer formation. This excellent achievement, particularly considering the recycled nature of the PVB used, is effectively demonstrated for the first time in this study.

## 3 Conclusion

As demand for PVB rises to produce laminated safety glasses, so does awareness of its environmental footprint. Recycling offers a solution, as well as repurposing/upcycling of its nonrecyclable fractions into the circular economy, which is vital, supporting waste reduction and sustainable resource use. Thus, with a focus on sustainability, cost‐effectiveness, safety, and scalability, a novel SPE was developed through a green, straightforward, and low‐cost solvent‐free process by blending PEO with PVB, particularly the nonrecyclable fraction derived from automotive glass waste. This approach enabled an enhancement of mechanical strength, by combining the robust mechanical framework of PVB with the ion‐conductive properties of PEO. The resulting PEO/PVB‐blend solid polymer electrolyte exhibited a markedly higher mechanic strength compared to pristine PEO, while maintaining a high ionic conductivity of 7.1 × 10^−4^ S cm^−1^ at 70 °C. The improved mechanical robustness promoted uniform lithium plating and stripping, thereby significantly enhancing the cycling stability and extending the lifespan of solid‐state lithium‐metal batteries. Assessment of the electrochemical behavior, by using high‐loading LiFePO_4_ cathode, confirmed the practical applicability of the optimized electrolyte. The system demonstrated high specific capacity, excellent coulombic efficiency, and excellent rate capability, even under demanding operating conditions, highlighting its ability to maintain performance at industrially relevant loadings.

Overall, this study not only presents a promising strategy for enhancing the electrochemical performance of solid‐state lithium batteries but also contributes to the advancement of environmentally responsible energy storage technologies through the integration of recycled materials into functional components.

## 4 Experimental Section

4.1

4.1.1

##### Reagents

Poly(vinylidene fluoride) (PVDF) was supplied by Solvay. Lithium bis(trifluoromethanesulfonyl)imide (LiTFSI), poly (ethylene oxide) (PEO, average molecular weight ≈ 4 000 000), PVB, conductive carbon (Super C65), N‐methyl‐2‐pyrrolidone (NMP), and lithium iron phosphate (LiFePO_4_, LFP) were sourced from Sigma‐Aldrich. Recycled PVB (Rec PVB) was derived from automotive glass waste using a patented mechanochemical method. Previous research employing FT‐IR, TGA, and NMR techniques has demonstrated that this recycling process preserves the molecular weight of the polymer (around 135 000 g mol^−1^, with a wide distribution) without significant alteration. Additionally, the analysis revealed the presence of several plasticizers, all from the diester group, with an approximate polymer‐to‐plasticizer weight ratio of 1:1.^[^ ^39^ ^]^ All reagents were used as received without further purification, except for the polymeric materials and LiTFSI salt that were preconditioned by vacuum drying at 50 °C overnight to remove residual moisture.

##### SPE Preparation

SPEs were prepared using a solvent‐free method. PEO was blended with LiTFSI salt in two molar ratios: 21:1 and 15:1. After achieving a homogeneous mixture, varying amounts of PVB (3, 5, and 10%) were incorporated into the mixture. The resulting composite was further homogenized using an agate mortar. The prepared mixtures were hot‐pressed between two Teflon disks at 70 °C under 2 tons of pressure, yielding uniform, self‐standing films. These films were cut into 16 mm disks and dried overnight at 50 °C under dynamic vacuum in a Büchi oven, then transferred to an argon‐filled glove box (Jacomex GP Campus with O_2_ and H_2_O levels < 0.8 ppm).

Two series of SPE were prepared; incorporating different molar ratios of EO to Li (21:1 and 15:1) and varying PVB contents (3%, 5%, and 10%) were labeled as 21‐3, 21‐5, and 21‐10 (Series 21), and 15‐3, 15‐5, and 15‐10 (Series 15), respectively. For comparison, a pristine SPE without PVB was also synthesized using the same protocol. Furthermore, the most promising membrane composition was utilized to develop a SPE in which commercial PVB was replaced with Rec PVB, designated as 15‐Rec 10.

##### Characterization and Measurements

XRD analysis was performed using a Rigaku diffractometer with Bragg–Brentano geometry and a Cu‐Kα X‐ray source (*λ* = 1.54059 Å), 2*θ* = 10° to 75°. The intensity of the spectra was normalized. SEM images were obtained using a Zeiss Sigma 300 FE‐SEM. A metallization step to form a 9 nm thick chromium layer was needed to improve the electrolyte conductivity. The thermal stability of the SPE was studied with a PerkinElmer STA6000 TGA‐DTA instrument between 30 and 550 °C under nitrogen flow and with a heating ramp of 10 °C min^−1^.

DSC analysis was performed using a Netzsch 214 Polyma instrument. The experiment was conducted within a temperature range of −80 to 120 °C at a heating rate of 10 °C min^−1^ under a nitrogen flow of 50 mL min^−1^. The degree of crystallinity was calculated from the integral area of the melting signal (ΔH_m_), compared with a melting enthalpy of 205 J g^−1^ for 100% crystalline PEO (ΔH_m_^0^).^[^ ^69^, ^70^ ^]^

The mechanical properties were investigated by using a Hysitron/Bruker Triboindenter TI 950 in displacement‐controlled mode. A three‐sided pyramidal Berkovich tip was used as the indenter. The maximum penetration depth was limited to 500 nm, corresponding to less than 10% of the film thickness, to minimize substrate effects^[^ ^71^ ^]^ For each material, 50 nanoindentations were performed. The spacing between two consecutives nanoindentations was 5 μm. The values of reduced Young's modulus were calculated from the unloading segment of the load‐displacement curves using the Oliver and Pharr method.^[^ ^72^ ^]^

EIS and galvanostatic cycling with potential limitation were conducted at 65 °C using a Li||SPE||Li symmetric cell to evaluate, respectively, the resistance and cycling performance of the membranes. The measurements were conducted using 12 mm electrodes and 16 mm electrolyte membranes in CR2032 coin‐type cells, assembled in an inert argon atmosphere. The Nyquist plots were modeled using the equivalent circuit method through a nonlinear least square fitting protocol with the RelaxIS 3 software (rhd instruments, Darmstadt, Germany). The employed equivalent circuit was R_e_(R_*i*_C_*i*_)Q_w_ according to Boukamp notation where R_e_ is the SPE resistance, R_*i*_ and C_*i*_ represent the interfacial resistance, due to charge‐transfer, and capacitance, due to double layer formation, Q_w_ describes the impedance due to semi‐infinite diffusion; however, to perform a more accurate fitting, the capacitive element (C_*i*_) was replaced with a constant‐phase element (Q), this accounting for surface irregularities or roughness. Lithium stripping and plating tests were evaluated over a minimum of 40 cycles at a current density of 0.1 mA cm^−2^. For the most promising SPE, the stability assessment was extended with additional cycles.

Ionic conductivity (σ) of the samples was assessed by EIS measurements spanning a frequency range of 0.1 Hz to 1 MHz with an applied 20 mV AC sinusoidal voltage. The experimental setup featured a SPE disc (16 mm in diameter, ≈150 μm thick) positioned between two stainless‐steel (SS) blocking electrodes, forming a SS||SPE||SS configuration within an EL‐Cell Std (EL‐CELL, Germany). To ensure precise determination of the cell constant, SPE thickness was accurately measured before and after each experiment using a micrometer (Mitutoyo). Experiments were conducted in a temperature‐controlled chamber (MK 53 E2, BINDER, Germany) over a T range of 0–80 °C. Prior to measurements, cells were equilibrated for 100 min at each temperature, with data collected at 10 °C intervals. *σ* was calculated using the equation: *σ* = L/(R × A), where L is the thickness of the SPE, R is the bulk resistance obtained from EIS, and A is contact surface area.

The ESW was investigated at T = 65 °C, in a cell configuration where the SPE was placed between a lithium disk and a working electrode (WE) made of conductive carbon and PVDF (8:2 weight ratio) casted onto an aluminum current collector, by: 1) anodic linear sweep voltammetry (LSV) from E = 3 V (OCV) to 5 V, with a scan rate of 0.1 mV s^−1^; 2) chronoamperometry steps at increasing potentials in the same 3 V to 5 V potential range.

To calculate the Li^+^ ion transference number (t_Li+_), a chronoamperometric measurement (applying a voltage of 30 mV for 90 min) together with EIS (frequency range from 100 kHz to 5 mHz) was performed on a Li||SPE||Li symmetric cell at two different temperatures of 55 and 65 °C. Li^+^ transference number was calculated by means of the Bruce–Vincent–Evans method.^[^ ^73^ ^]^

To evaluate the practical application potential of the SPEs in a real cell configuration, laboratory scale solid state Li‐metal cells were assembled in a Li||electrolyte||LFP (catholyte) configuration using an EL‐Cell Std (EL‐CELL, Germany) for galvanostatic cycling tests at 65 °C under different current densities (C‐rate capability) by starting at C/40 and increasing every five cycles up to C/2 (C/40, C/20, C/10, C/5, and C/2), and back to C/40. In this protocol, 1 C was assumed to correspond to 170 mA g^−1^ with respect to the active material mass in the LFP‐based electrode. Cathode laminates were fabricated using a composite mixture consisting of commercial LiFePO_4_ (Alfa Aesar), carbon black (C65, TIMCAL), and a catholyte with the same composition as the PEO–LiTFSI electrolyte. The components were combined in a weight ratio of 75:5:20 (LFP:C65:catholyte). Prior to mixing, the LFP and C65 powders had been dried under vacuum at 80 °C for 12 h to remove residual moisture. The resulting slurry was then cast onto aluminum foil using a doctor blade with a 500 μm gap and subsequently dried under vacuum at 80 °C overnight.

All electrochemical measurements have been performed by using a VMP3 potentiostat/galvanostat (Biologic).

## Conflict of Interest

The authors declare no conflict of interest.

## Supporting information

Supplementary Material

## Data Availability

The data that support the findings of this study are available from the corresponding author upon reasonable request.
