# Supplementary material for: Unlocking Sustainable‐by‐Design Li‐Metal Batteries by Recycled PVB in Blend Polymer Electrolytes
Source: ChemSusChem. 2025 Aug 18;18(21):e202501288. doi: 10.1002/cssc.202501288 (PMC12584968; doi:10.1002/cssc.202501288)
Supplement: Supplementary file 1 — Supplementary Material [file CSSC-18-e202501288-s001.pdf]

Unlocking sustainable-by-design Li-Metal Batteries by recycled PVB in blend polymer electrolytes

Asia Patriarchi[a], Hamideh Darjazi[b,c]*, Alessandro Piovano[b,c], Leonardo Balducci[a,b], Nicolò Arcieri[b], Miguel Ángel Muñoz[a,c], Francesco Nobili[a,c], Claudio Gerbaldi[b,c]

[a]*School of Science and Technology; Chemistry division, University of Camerino, Via Madonna delle Carceri-ChIP, Camerino (MC) 62032, Italy*

[b]*Department of Applied Science and Technology, Politecnico di Torino, Corso Duca degli Abruzzi, 24, 10129, Torino, Italy*

[c]*National Reference Center for Electrochemical Energy Storage (GISEL) - INSTM, Via G. Giusti 9, Firenze 50121, Italy.*

Corresponding author: Hamideh Darjazi (hamideh.darjazi@polito.it)

Supplementary information

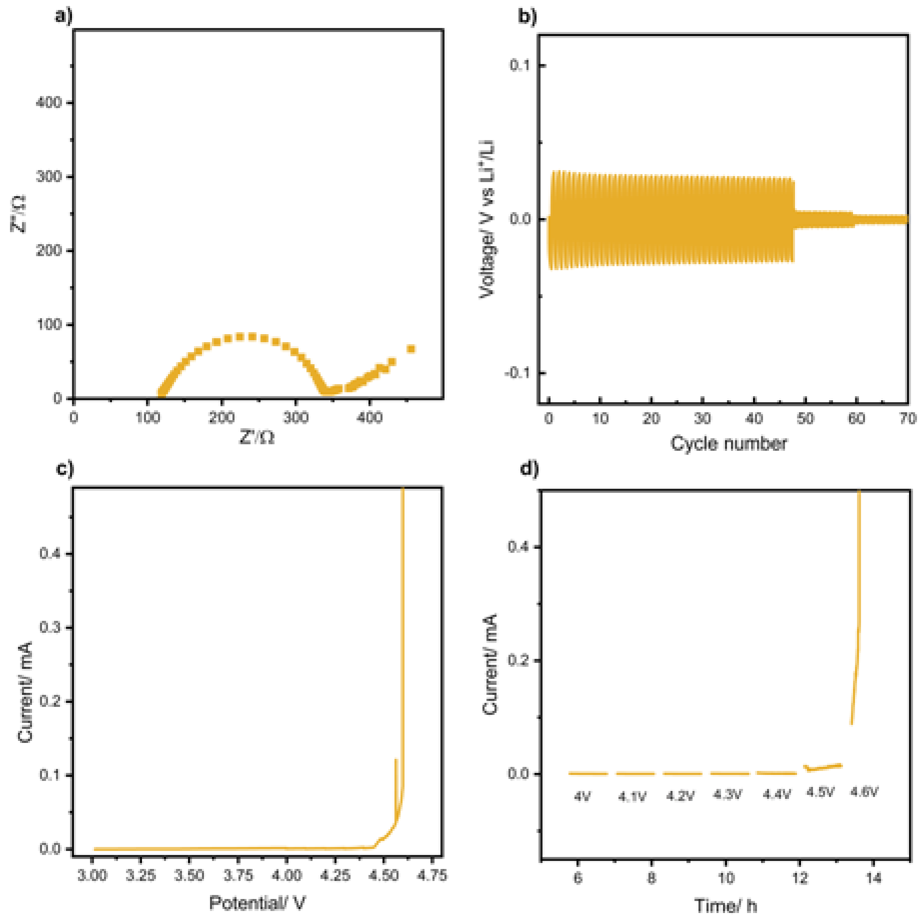

Figure S1: Nyquist plots (a) and lithium stripping/plating tests recorded at 65°C in the Li||SPE||Li symmetric cells for pristine membrane. (b). LSV (c) and CA curves (d) of the WE||SPE||Li cell at 65°C.

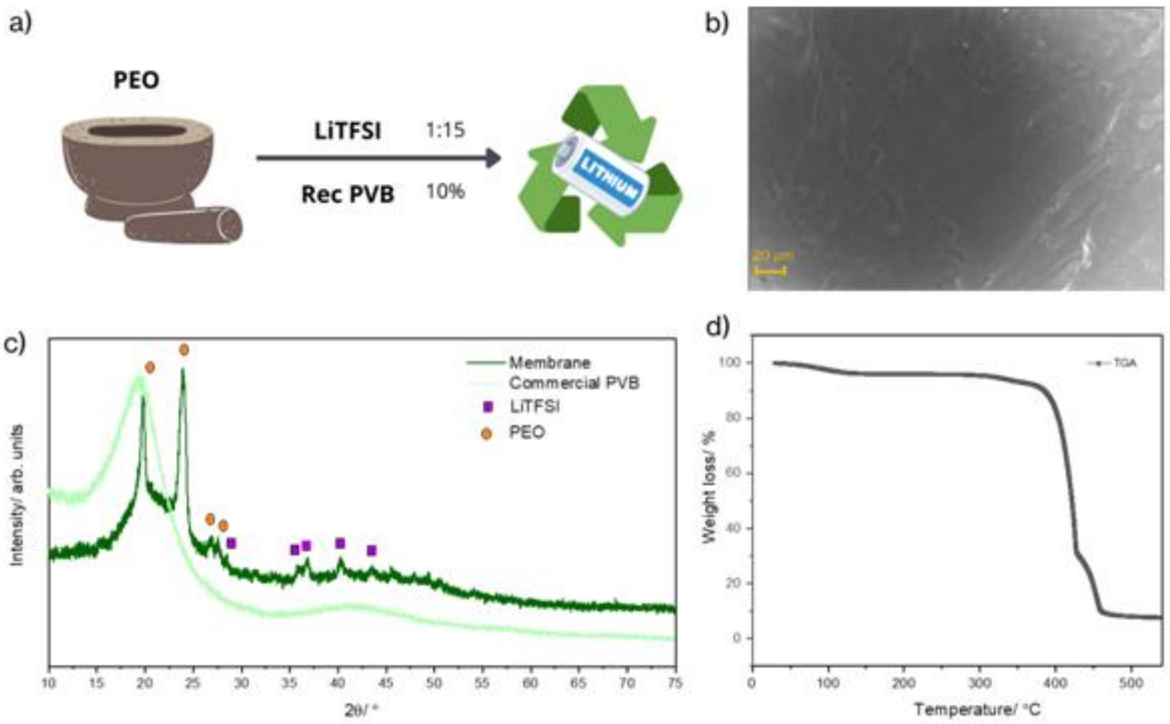

Figure S2: schematic representation of the synthetic procedure used for the synthesis of the SPE incorporating 10% of Rec PVB (a). Electrolyte surface overview image obtained by SEM analysis(b). XRD diffraction pattern obtained of 15-Rec 10 overlapped with commercial PVB pattern (c). TGA curves in the 30-550°C under nitrogen flow (d).

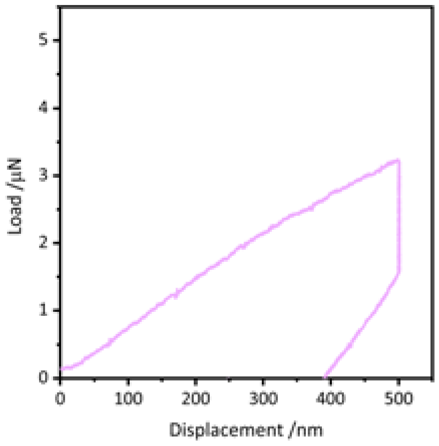

Figure S3. Nanoindentation curve for 15-10 membrane.

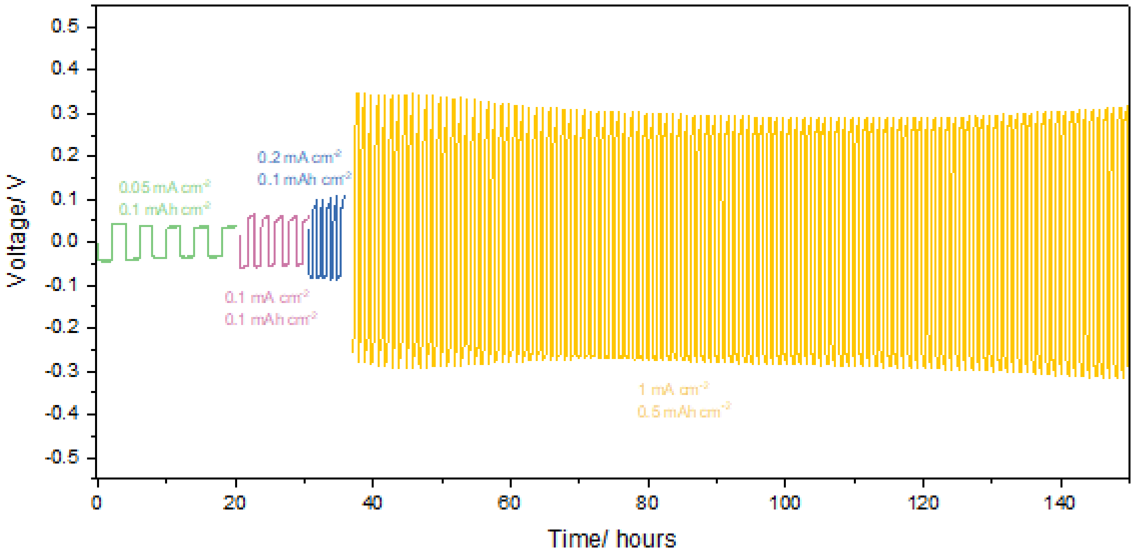

Figure S4. Stripping/plating tests conducted using the 15Rec 10 electrolyte in Li|Li symmetric cell at various current densities.

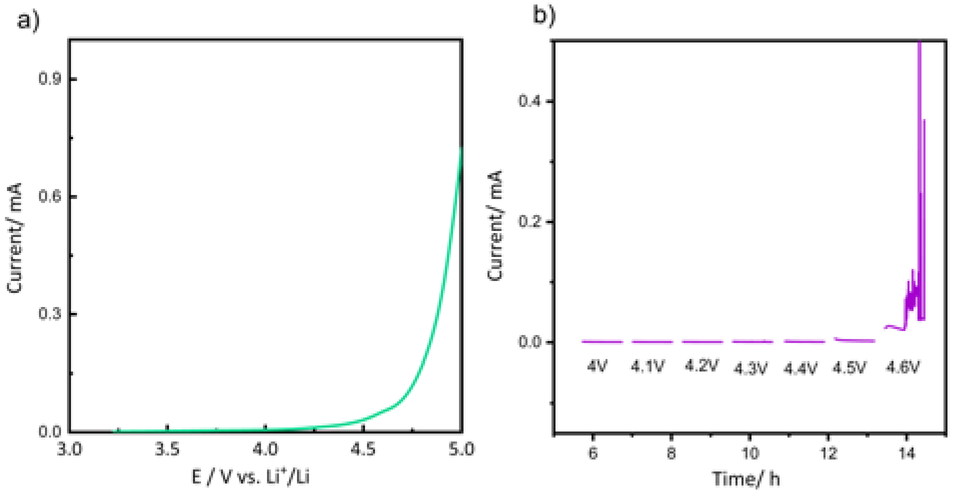

Figure S5. LSV (a) and CA curves (b) of the WE||SPE||Li cell at 65℃.

Table S1: Comparison of previously reported SPEs in lithium batteries.

Electrolyte	***Cathode and mass loading***	***C rate***	***Specific capacity***	***Ref***
LiTFSI-PEO-0.2AlF_3	LFP, 2 mg cm^{-2}	50 mA g^{-1}	167.4 mAh g^{-1}	(Hu et al., 2022)
PPS-SPE	LFP, 1.9 mg cm^{-2}	0.2 C	139.5 mAh g^{-1}	(Zhang et al., 2017)
PEO/LiTFSI SPE	LFP, not reported	0.3 C	157.5 mAh g^{-1}	(Li et al., 2022)
PEO/LiTFSI/MnO_2 CSPE	LFP, 1.3 mg cm^{-2}	0.5 C	143.5 mA h g^{-1}	(Li et al., 2020)
PEO-LiTFSI-20LAHP	LFP, 4 mg cm^{-2}	50 μA cm^{-2}	155 mA h g^{-1}	(Zhou et al., 2021)
15-Rec10	LFP, 13 mg cm^{-2}	0.025 C	169.8 mAh g^{-1}	This work

Bibliography

1. Hu J, Lai C, Chen K, Wu Q, Gu Y, Wu C, et al. Dual fluorination of polymer electrolyte and conversion-type cathode for high-capacity all-solid-state lithium metal batteries. Nat Commun. 2022;13(1).

2. Zhang D, Zhang L, Yang K, Wang H, Yu C, Xu D, et al. Superior Blends Solid Polymer Electrolyte with Integrated Hierarchical Architectures for All-Solid-State Lithium-Ion Batteries. ACS Appl Mater Interfaces. 2017;9(42).

3. Li Y, Fu Z, Lu S, Sun X, Zhang X, Weng L. Polymer nanofibers framework composite solid electrolyte with lithium dendrite suppression for long life all-solid-state lithium metal battery. Chemical Engineering Journal. 2022;440.

4. Li Y, Sun Z, Liu D, Gao Y, Wang Y, Bu H, et al. A composite solid polymer electrolyte incorporating MnO2 nanosheets with reinforced mechanical properties and electrochemical stability for lithium metal batteries. J Mater Chem A Mater. 2020;8(4).

5. Zhou Q, Li Q, Liu S, Yin X, Huang B, Sheng M. High Li-ion conductive composite polymer electrolytes for all-solid-state Li-metal batteries. J Power Sources. 2021;482.
